# Stimulation parameters of epidural spinal cord stimulation for functional recovery after spinal cord injury: a narrative review

**DOI:** 10.3389/fneur.2026.1952514

**Published:** 2026-10-19

**Authors:** Jingkai Wang, Yermike Bieshanbieke, He Wang, JiChao Yang, Shanfeng Ji, YuLong Li, HaoYuan Gu, YongLi Lou

**Affiliations:** 1 Henan Medical University, Xinxiang, China; 2 Zhengzhou Central Hospital Affiliated to Zhengzhou University, Zhengzhou, China; 3 Xinjiang Medical University Affiliated Second Hospital, Ürümqi, China

**Keywords:** epidural spinal cord stimulation, functional recovery, neuromodulation, spinal cord injury, stimulation parameters

## Abstract

Spinal cord injury (SCI) causes persistent motor, sensory, and autonomic deficits that conventional rehabilitation only partly mitigates. Epidural spinal cord stimulation (EES) can re-enable standing, walking, upper-limb control, and autonomic regulation, yet clinical translation is constrained by pronounced heterogeneity in stimulation parameters and by a weakly controlled evidence base. This narrative review maps stimulation parameters—electrode placement, frequency, intensity, pulse width, waveform, and stimulation mode—onto functional targets after SCI, critically appraises the supporting evidence, and maps scenario-specific parameter considerations onto a descriptive evidence hierarchy. Evidence was identified through targeted, non-systematic searches of PubMed/MEDLINE and citation tracking of key primary studies and reviews through 30 June 2026; human epidural-stimulation studies in SCI reporting parameters or functional outcomes were prioritized, with preclinical studies retained for mechanistic synthesis, transcutaneous trials used only as contextual evidence, and preprints labeled as such. Because no systematic search was performed, the review is presented as a narrative review: evidence is appraised using a design-based hierarchy, human, animal, and preprint findings are explicitly distinguished, and all parameter-level considerations are offered as preliminary observations rather than graded recommendations. Two reproducible motor parameter philosophies emerge—tonic, individually titrated lumbosacral stimulation embedded in intensive activity-based training, and programmed biomimetic (spatiotemporal, dual-mode, closed-loop) approaches—whereas hemodynamic management now has the most standardized parameter set. Evidence for upper-limb, bladder, spasticity, and pain indications remains case-level, and for neuropathic pain no SCI-specific parameter evidence exists. Mechanistic evidence derives predominantly from animal models, and clinical reports are dominated by small uncontrolled series in which stimulation is delivered as one component of a combined stimulation-plus-training intervention. Parameter-level conclusions are therefore preliminary and hypothesis-generating rather than practice-defining; standardized parameter reporting, physiology-based stratification, and adequately powered controlled trials are the priorities for converting EES from individualized experimentation into evidence-based neurorehabilitation.

## 1 Introduction

Spinal cord injury (SCI) is a life-altering neurological condition that disrupts communication between the brain and the body, producing permanent or severe motor, sensory, and autonomic impairments that profoundly diminish independence and quality of life (1, 2). Globally, the incidence of SCI is approximately 23.77 per million people, with traumatic SCI predominating, occurring more frequently in males, and increasing with age (3). An initial mechanical insult is followed by a protracted secondary injury cascade—ischemia, excitotoxicity, oxidative stress, inflammation, demyelination, and glial scar formation—that expands the lesion and constrains endogenous repair (1, 2, 4, 5). Despite decades of intensive research, conventional rehabilitation strategies, while essential for maximizing residual function, yield limited functional restoration in individuals with severe or chronic SCI, underscoring the need for interventions that can augment the intrinsic recovery capacity of the spinal cord (2, 6).

Epidural spinal cord stimulation (EES) has emerged at the forefront of this effort. By delivering electrical pulses to the dorsal surface of the spinal cord, EES recruits large-diameter sensory afferents and raises the excitability of spinal motor networks below the lesion, enabling residual descending commands to elicit motor output even in individuals classified as motor-complete (7–9). Landmark demonstrations have shown that EES, typically combined with intensive activity-based training, can restore weight-bearing standing and volitional leg movement after motor-complete paraplegia (8, 10), over-ground walking after chronic incomplete injury (11, 12), and elements of autonomic regulation, including bladder, cardiovascular, and sexual function (7, 13). However, the pronounced heterogeneity of SCI—in injury level, severity, chronicity, and residual anatomy—means that these outcomes were achieved with highly individualized stimulation configurations, and the process of selecting parameters in most studies has remained empirical, iterative, and sparsely reported.

Yet the efficacy of EES is critically dependent on stimulation parameters—electrode placement and configuration, frequency, intensity, pulse width, waveform, and temporal mode—and the field's evidence base is too fragmented to support definitive parameter guidance. A scoping review of 103 human studies of spinal cord stimulation for SCI found that case studies and series predominated, only one randomized controlled trial (RCT) had been conducted up to March 2021, 70% of studies enrolled 10 or fewer participants, and stimulation parameters were incompletely described across many reports (14). A subsequent appraisal restricted to epidural stimulation identified only 13 eligible case series totaling 88 participants, noted that no formal risk-of-bias assessment was possible, and cautioned that every included publication was supportive of the intervention, with no reports of significant failures—a pattern strongly suggestive of reporting and publication bias (15). A systematic review of 64 human EES studies encompassing 306 patients similarly documented pronounced device and parameter heterogeneity and called explicitly for standardized stimulation parameters (16). Compounding this fragmentation, reported “optimal” frequencies span two orders of magnitude across studies, amplitude is reported in incompatible units (volts vs. milliamperes), and pulse width is frequently omitted altogether, preventing any defensible cross-study dose comparison (14, 15).

This fragmentation is particularly consequential because the field is advancing rapidly. Closed-loop brain–spine interfaces that decode cortical motor intent to modulate EES in real time have restored natural walking in a first-in-human proof of concept (17), extending earlier non-human primate work (18). Bidirectional perilesional stimulation now pairs sublesional motor EES with supralesional sensory-feedback stimulation, using deep-learning optimization to individualize parameters (19). Mechanistic work has redefined how residual descending input interacts with subthreshold stimulation to bound the “functional parameter space” as a function of lesion severity (20). Autonomic targeting has matured from anecdote to systematic synthesis, with parameters now being mapped explicitly to cardiovascular, bladder, bowel, and sexual outcomes (21–23), and a transcutaneous spinal stimulation system has received the first regulatory authorization in this therapeutic class on the basis of a single-arm pivotal trial (24). Existing reviews predate or only partially capture these developments.

Against this background, the present narrative review aims to provide a parameter-focused, decision-oriented synthesis of EES for functional recovery after SCI. Specifically, we (1) critically summarize the rationale and neural mechanisms of EES, distinguishing animal-derived mechanistic claims from human evidence; (2) synthesize reported stimulation parameters and their functional consequences across electrode placement, frequency, intensity, pulse width, waveform, and stimulation mode; (3) appraise the evidence hierarchy, methodological heterogeneity, and unresolved contradictions that complicate cross-study comparison; and (4) map scenario-specific parameter considerations and descriptive evidence levels, presented as preliminary observations rather than graded recommendations, organized by clinical goal (standing, walking, upper-limb function, spasticity, and autonomic targets). In doing so, this review responds directly to the field's central problem: an expanding but fragmented literature in which uniformly positive uncontrolled reports coexist with incompatible parameter conventions. Rather than adding another descriptive catalog, we aim to equip clinicians and investigators with a critical framework for interpreting parameter choices, recognizing the limits of the current evidence, and prioritizing the study designs needed to resolve it. Throughout, we abbreviate epidural stimulation as EES (the same intervention is denoted scES or eSCS in parts of the cited literature) and transcutaneous spinal cord stimulation as tSCS.

## 2 Review approach: evidence identification, appraisal, and synthesis

### 2.1 Review design

This article is a narrative review. In response to the previous round of peer review, the manuscript has been reclassified from a scoping review to a narrative review, consistent with the editorial office's guidance, because the literature was identified through targeted, non-systematic searching rather than a protocol-driven systematic search. Reporting frameworks for scoping reviews (25, 26) presuppose a protocol-driven systematic search; because none was performed, those frameworks are not applicable: no review protocol was registered, no PRISMA-ScR checklist applies, and no formal screening log or quantitative study-selection flow diagram is presented. A narrative design is appropriate for our aims—thematic synthesis across parameters, outcomes, mechanisms, and evidence quality in a literature dominated by small, heterogeneous, uncontrolled studies that preclude meaningful meta-analysis (14, 16)—provided that the identification process is described transparently and that parameter-level statements are framed as preliminary observations rather than graded recommendations; both conventions are followed throughout this article.

### 2.2 Evidence identification

Targeted searches of PubMed/MEDLINE were performed through 30 June 2026 using combinations of terms for spinal cord injury (“spinal cord injur^*^”) and epidural stimulation (”epidural spinal cord stimulation,” “epidural electrical stimulation,” “spinal cord epidural stimulation,” scES, eSCS, EES), complemented by backward and forward citation tracking from key primary studies and from recent systematic and scoping reviews of the field (14–16, 21, 23). Records were prioritized when they (i) reported human epidural-stimulation studies in SCI with stimulation parameters and/or functional outcomes in any domain (motor, sensory, autonomic, spasticity, pain); (ii) were preclinical studies in rodent, feline, or non-human primate models informing mechanisms or parameter rationale; (iii) were systematic or scoping reviews used for contextual and parameter-range synthesis; or (iv) were key transcutaneous spinal cord stimulation (tSCS) trials bearing on the evidence hierarchy or clinical translation (e.g., sham-controlled or regulatory evidence). Transcutaneous evidence is explicitly identified wherever cited and was used only as contextual evidence, never as direct evidence of epidural efficacy (Section 5.4c). Preprints reporting otherwise unavailable clinical data were eligible and are labeled as such throughout. Conference abstracts without extractable parameter data and studies of spinal stimulation for indications unrelated to SCI were excluded.

### 2.3 Evidence appraisal and synthesis

Because no validated risk-of-bias tool applies to case reports and uncontrolled series, and formal risk-of-bias assessment has been judged infeasible in this literature (15), sources are characterized using a design-based hierarchy adapted from the Oxford Center for Evidence-Based Medicine levels of evidence (27): moderate-low for controlled but non-randomized cohorts; low for uncontrolled case series; and very low for case reports, preprints, and preclinical-only evidence. The evidence levels shown in Tables 1, 2 were assigned by applying this scheme to the most rigorous available study design supporting each row; they are descriptive aids to interpretation, not formal evidence gradings. Within both tables, human studies, animal studies, and preprints are distinguished explicitly in the evidence columns. Given the predominance of small, uncontrolled studies, the parameter ranges in Table 1 and the clinical takeaways throughout are presented as preliminary, hypothesis-generating observations—not practice-defining recommendations. Synthesis was narrative and scenario-based: parameters were mapped onto clinical goals (standing, walking, upper-limb function, spasticity, bladder and bowel, cardiovascular regulation, and pain), and convergences, contradictions, and reporting gaps were identified across sources (Tables 1, 2).

**Table 1:** Stimulation parameter ranges by clinical scenario, with representative evidence and evidence level.

Clinical scenario	Electrode target	Frequency	Intensity	Pulse width	Mode	Representative evidence	Evidence level
Standing	Lumbosacral paddle, T11–L1 vertebral (L1–S1 segments); caudal-cathode configurations	15–60 Hz (25–60 Hz typical)	1–9 V constant voltage, near motor threshold to suprathreshold	210–450 μs (center practice; often not reported)	Tonic, individually optimized, + weight-bearing training	Harkema et al. (8) (human); Rejc et al. (10) (human); Angeli et al. (33) (human); Rybka et al. (65) (human); Chhabra et al. (42) (human)	Case series/case reports (*n* = 1–10 per cohort)
Walking/locomotion	Lumbosacral paddle T11–L1, or T12–S1 over posterior roots	Tonic 15–50 Hz; or per-program spatiotemporal ~20–120 Hz (flexion-biased ~40–120 Hz; extensor-biased ~20–30 Hz)	Individually optimized (V or mA; program-specific)	210–450 μs (tonic); ~500 μs (spatiotemporal programs)	Tonic + months of activity-based training; or gait-phase-triggered spatiotemporal programs	Angeli et al. (11) (human); Wagner et al. (12) (human); Gill et al. (41) (human); Kathe et al. (28) (human); Rowald et al. (61) (human); Wan et al. (66) (human); Ren et al. (67) (human)	Case series (*n* = 3–9) plus one non-randomized cohort (*n* = 21); no randomized trials
Upper-limb/hand function	Cervical enlargement (C4–T2), dorsal cervical paddle caudal to lesion	Mapping 2–100 Hz; flexor-biased 10–30 Hz, extensor-biased 40–70 Hz	0.1–20 mA constant current (~0.5–3 × motor threshold in functional use)	100–500 μs	Task-specific tonic; location/intensity/frequency-optimized phase-switching programs	Oh et al. (44) (human, preprint); Greiner et al. (37) (animal, NHP); Barra et al. (43) (animal, NHP)	Case-level clinical (*n* = 2, preprint) + preclinical
Spasticity	Lumbar/lumbosacral (T10–L3); dual-mode: lesion-proximal/lumbosacral paddles	Tonic 50–130 Hz (historical); or kilohertz-range HF block (e.g., 1.2 kHz) paired with low-frequency (30–60 Hz) facilitation	1.5–4 V (tonic); individually titrated (dual-mode)	120–300 μs (tonic); program-specific (dual-mode)	Chronic tonic; or dual-mode HF + LF with rehabilitation	Pinter et al. (72) (human); Dekopov et al. (52) (human); Romeni et al. (51) (human); Fan et al. (47) (human, preprint)	Case series (*n* = 2–31); no dose-finding trials
Bladder/bowel	Lumbosacral paddle T11–L1 (L1–S1 segments); caudal-contact weighting	~30 Hz for voiding; bladder-targeted mapping programs up to 60–90 Hz	Individually set	450 μs typical (300–1,000 μs mapped)	Tonic bladder-targeted configurations during/after mapping	Herrity et al. (62) (human); Herrity et al. (63) (human); Darrow et al. (7) (human)	Case series (*n* = 2–10, single center); bowel: secondary outcomes only
Blood pressure/ cardiovascular	Lower-thoracic hemodynamic hotspot (last three thoracic segments); earlier studies used lumbosacral arrays	120 Hz (hotspot-targeted); 2–35 Hz individually optimized (lumbosacral)	≤ 20 mA, titrated to pressor response or comfort limit	~300 μs (100–500 μs mapped); 450 μs (lumbosacral)	Continuous or closed-loop (PID/neuroprosthetic baroreflex)	Phillips et al. (56) (human); Squair et al. (55) (human); Aslan et al. (58) (human + preclinical)	Clinical case series (*n* = 14 across four trials) + preclinical; most standardized parameter set
Neuropathic pain	Dorsal columns above/at lesion (cervical or thoracic)	Tonic 30–80 Hz; high-frequency 1–10 kHz (sub-perception); burst (40-Hz bursts of 500-Hz spikes)	Paresthesia-level (tonic); sub-perception (HF/burst)	100–500 μs (tonic); ~30 μs (10-kHz HF); 120–800 μs (1.2-kHz HF); 1 ms (burst spikes)	Conventional pain-SCS paradigms; no SCI-specific optimization	Joosten and Franken (64) (review); Sokal et al. (60) (human); Dombovy-Johnson et al. (59) (human)	Case series only (2/8 responders at 12 months); systematic-review GRADE: low; no RCT

Amplitudes reported in volts derive from constant-voltage devices and are not directly comparable with milliamperes from constant-current devices; values must be interpreted within their original unit system. All parameter values are ranges reported in the cited studies (or extracted in peer-reviewed systematic-review parameter tables) and are provided for reference and hypothesis generation, not as prescription-level recommendations; all listed protocols were individually optimized. NR = not reported in the source; PW values marked “center practice” reflect the implanting center's standard settings rather than values stated in the paper. Preprints are identified as such and have not completed peer review. Evidence levels follow the design-based scheme defined in Section 2.3; human, preclinical, and preprint evidence is distinguished in the Representative evidence column.

Hz, hertz; μs, microseconds; V, volts (constant-voltage devices); mA, milliamperes (constant-current devices); AIS, American Spinal Injury Association Impairment Scale grade; HF, high frequency; NHP, non-human primate; PID, proportional–integral–derivative; RCT, randomized controlled trial.

**Table 2:** Evidence hierarchy and consistency of findings by outcome domain for EES after spinal cord injury.

Outcome domain	Representative studies	Design	*N*	Evidence level	Consistency of findings
Standing	Harkema et al. (8); Rejc et al. (10); Angeli et al. (33); Rybka et al. (65); Chhabra et al. (42) (all human)	Case report/uncontrolled case series	1–10 per cohort	Low (case series, no controls)	Consistent direction across ≥3 independent centers, but uncontrolled; effects largely stimulation-dependent
Walking/ locomotion	Angeli et al. (11); Wagner et al. (12); Kathe et al. (28); Rowald et al. (61); Wan et al. (66); Ren et al. (67) (all human)	Case series; one non-randomized controlled cohort	1–9; Ren et al. (67): 21	Low; moderate-low for Ren (67) (non-randomized)	Consistent but uncontrolled; over-ground walkers predominantly sensory-spared/incomplete; gains accrue to the combined stimulation-plus-training intervention; training dose and protocol vary widely across studies (Section 5.4e)
Upper-limb (cervical EES)	Oh et al. (44) (human, preprint); Lu et al. (96) (human); Greiner et al. (37) (animal, NHP)	Case reports; preclinical	2 (single clinical study)	Very low (case-level)	Sparse; mechanistically coherent; no controlled human trial (largest adjacent trial is transcutaneous: (24), single-arm)
Trunk/postural control	Joshi et al. (45); Rejc et al. (69); Rowald et al. (61) (all human)	Pilot studies	3–9	Very low	Conflicting; gains are task- and program-specific, no transfer between standing and sitting
Sensory function	Ren et al. (67) (human); Solinsky et al. (70) (human); Fan et al. (47) (human, preprint)	Non-randomized controlled cohort; retrospective; preprint	21 (67)	Low	Consistent direction but secondary endpoints only; gains cluster near the neurological level of injury
Bladder/bowel	Herrity et al. (62); Herrity et al. (13); Herrity et al. (63); Rybka et al. (65); Darrow et al. (7) (all human)	Single-center case series (urodynamic)	2–10	Low	Consistent for storage/voiding parameters (~30 Hz voiding; mapping up to 60–90 Hz unreconciled); bowel evidence sparse and self-reported
Blood pressure/ cardiovascular	Aslan et al. (58) (human + preclinical); Squair et al. (55) (human); Phillips et al. (56) (human); Samejima1 (human); Upadhyay et al. (23) (review)	Mechanistic + multi-trial case series; systematic review	1–14	Low-moderate (convergent, mechanism-anchored)	Consistent; most standardized parameter set of any domain (hotspot at the last three thoracic segments, 120 Hz, closed-loop); still uncontrolled
Spasticity	Pinter et al. (72) (human); Dekopov et al. (52) (human); Romeni et al. (51) (human); Fan et al. (47) (human, preprint); Jung et al. (73) (review)	Retrospective series; systematic review of low-quality studies	2–31	Low (review-grade: low quality, high heterogeneity)	Consistent subjective improvement, but paradigms conflict (tonic 50–130 Hz vs. kHz-range conduction block); no dose-finding trials
Neuropathic pain	Sokal et a. (60); Dombovy-Johnson et al. (59)^;^ Ren et al. (67) (all human)	Case series; systematic review (69 pooled patients)	8; 69 (pooled)	Very low (GRADE low; no RCT)	Mostly negative, especially in complete SCI (2/8 responders at 12 months); parameters borrowed from pain practice

Evidence levels were assigned using the design-based scheme defined in Section 2.3; preprints and preclinical studies are labeled as such, and N values denote participants per cohort unless otherwise stated. NHP, non-human primate; GRADE, Grading of Recommendations Assessment, Development and Evaluation; RCT, randomized controlled trial.

## 3 Rationale and neural mechanisms of EES

The therapeutic rationale for EES rests on the observation that the sublesional spinal cord retains substantial intrinsic processing capacity after SCI, and that this capacity can be accessed and shaped by electrical stimulation. This section first summarizes the pathophysiological substrate and then critically examines the mechanisms proposed to underlie EES-mediated recovery, with explicit attention to the species from which each line of evidence derives.

### 3.1 Pathophysiology of SCI: the substrate for neuromodulation

SCI begins with a primary mechanical insult—contusion, compression, or transection—that destroys neural tissue and disrupts ascending and descending axonal pathways (1). This triggers a secondary cascade of ischemia, excitotoxicity, oxidative stress, inflammation, demyelination, and glial scar formation that unfolds over days to weeks, enlarges the lesion, and establishes a microenvironment hostile to axonal regeneration (1, 2, 4, 5). Functionally, the critical consequence is the loss of supraspinal input to spinal networks below the lesion. Importantly, however, these sublesional networks (including interneuronal circuits and central pattern generators capable of producing rhythmic motor output) often remain anatomically present but functionally dormant (9, 18, 28). Two further observations frame the rationale for EES. First, clinical classifications of “complete” injury do not necessarily imply anatomical completeness: the concept of “discomplete” SCI, in which subclinical brain influence persists across the lesion, predates EES by decades (29), and MRI-documented spared tissue at the lesion site predicts responsiveness to stimulation (30). Second, conventional rehabilitation alone rarely restores function when descending pathways are severely compromised (2, 6). EES is therefore conceived not as a regenerative therapy but as a means of re-engaging preserved circuitry—an interpretive frame that, as discussed below and in Section 5, has direct implications for how “recovery” under stimulation should be attributed.

### 3.2 Neural mechanisms of EES-mediated functional recovery

#### 3.2.1 Modulation of network excitability and activity-dependent plasticity

The canonical mechanism of EES is the recruitment of large-diameter proprioceptive afferents within the dorsal columns and dorsal roots, which project monosynaptically and polysynaptically onto spinal interneurons and motoneurons (9, 31, 32). The resulting afferent volley raises the excitability of spinal motor pools, lowering the threshold for motoneuron firing so that weak residual descending commands can be transformed into functional motor output (7–9). Recent translational work has refined this account substantially. Combining human electromyography (EMG) with non-human primate and computational data, Balaguer et al. (20) showed that EES pulses delivered at intensities subthreshold for direct motoneuron activation do not simply add background excitation: they become suprathreshold—and entrain motoneuron discharge in a time-locked manner—only when residual descending input depolarizes the motoneuron membrane into a permissive range. Stimulation-evoked afferent volleys and voluntary descending drive therefore interact multiplicatively rather than additively: the same pulse train may remain subthreshold, or may be converted into volitional motor output, depending on the state of residual descending excitation. This interaction defines a restricted “functional parameter space”—the set of amplitude, frequency, and placement combinations that permit such conversion—which narrows further as lesion severity increases (20). This model has direct parameter implications: it predicts that no universal parameter set exists, and that the effective window is an individual, anatomy-dependent quantity. Beyond immediate facilitation, EES combined with activity-based training can induce activity-dependent plasticity that persists after stimulation is switched off, presumably through reorganization and strengthening of residual corticospinal and intraspinal connections (33, 34); device-independent neurological recovery after brain–spine interface rehabilitation provides a notable recent example of such carry-over (17).

#### 3.2.2 Proprioception: facilitator and casualty of stimulation

Proprioceptive feedback is both the principal entry point of EES and its potential casualty. Computational, preclinical, and human data indicate that continuous EES can block proprioceptive input in humans—producing a transient deafferentation that abolishes leg-position perception and prevents the natural modulation of locomotion—whereas burst and spatiotemporal stimulation profiles mitigate this interference (31). In rats, spatiotemporal neuromodulation engaging muscle synergies outperformed continuous stimulation for locomotion (35), and spatiotemporal programs enabled adaptive walking in humans with motor-incomplete SCI (12). This evidence has been influential in motivating biomimetic, spatially selective stimulation paradigms. However, it sits uneasily with an equally robust clinical observation: continuous, tonic lumbosacral stimulation produced the field's most-cited functional gains in motor-complete cohorts, including full weight-bearing standing and volitional leg movement in all 13 participants of one series (10, 11, 30). Several factors may reconcile—or perpetuate—this contradiction: the Formento cohorts were largely motor-incomplete, in which preserved proprioception is worth preserving, whereas standing and single-joint volitional tasks in complete injury may depend less on continuous sensory modulation; species differences in dorsal-column geometry limit extrapolation of the rat data; and outcome type (rhythmic locomotion vs. static standing) interacts with stimulation mode. We flag this controversy here as a recurring theme, and return to its parameter-level consequences in Sections 4, 5.

#### 3.2.3 Recruitment of specific neuronal populations

EES does not act on the spinal cord as a homogeneous substrate. Single-nucleus transcriptomic and functional studies in mice identified a population of excitatory interneurons in the intermediate laminae of the lumbar cord whose activity is necessary and sufficient for walking recovery after paralysis: augmenting their activity mimicked EES-enabled recovery, while their ablation prevented it (28). Related work shows that neurotransmitter phenotype switching of spinal excitatory interneurons after adult injury biases the network toward inhibition and impairs locomotor recovery, whereas maintaining an excitatory phenotype promotes it (36). In non-human primates, cervical EES selectively recruited upper-limb motoneurons via sensory afferents, with recruitment modulated during movement, establishing a mechanistic basis for restoring arm and hand function (37). These findings imply that electrode placement and stimulation parameters should be understood as tools for biasing recruitment toward recovery-organizing neuronal subpopulations. They also carry an important translational caveat: interneuron-level specificity has been demonstrated only in rodent and primate models, and no human study has yet confirmed that clinical EES paradigms engage the homologous populations.

#### 3.2.4 Modulation of autonomic function

Autonomic dysfunction—including neurogenic bladder and bowel, cardiovascular instability, and sexual dysfunction—is pervasive after SCI and is increasingly recognized as a therapeutic target of EES in its own right. In individuals with chronic neurologically complete SCI, EES facilitated immediate re-engagement of dormant motor and autonomic supraspinal pathways, improving bladder–bowel synergy, cardiovascular regulation, and, in one participant, sexual function (7). Activity-based training combined with EES improved bladder storage parameters, although blood-pressure elevation during bladder distension persisted, motivating multi-target stimulation strategies (13). Preclinical mapping studies in decerebrate cat and rat models have localized detrusor activation to upper- and lower-lumbar stimulation and external urethral sphincter activation to sacral stimulation, providing a segmental rationale for site-specific autonomic neuromodulation (38, 39). More recently, lumbosacral EES—positioned conventionally for motor targets—was shown to acutely blunt autonomic dysreflexia in a case series, suggesting that motor-targeted implants can be re-parameterized for autonomic control (22). Two 2026 reviews have consolidated this literature, mapping stimulation parameters to cardiovascular, bladder, bowel, and sexual outcomes while emphasizing that the evidence remains dominated by case reports and small series (21, 23).

#### 3.2.5 Closed-loop control and brain–spine interfaces

Because SCI fundamentally disrupts brain-to-cord communication, a logical extension of EES is to restore that link artificially. In non-human primates, a brain–spine interface decoding leg motor-cortical activity to trigger epidural stimulation restored weight-bearing locomotion after SCI (18), and in rats with cervical SCI, cortical local-field-potential decoding of forelimb intent driving spinal stimulation improved forelimb function (40). This architecture has now reached humans: a fully implanted brain–spine interface linking cortical decoding to analog modulation of lumbosacral EES restored natural standing, walking, and stair climbing, with calibration stable over more than a year of home use and neurological recovery persisting with the device off (17). Bidirectional perilesional stimulation represents the complementary advance: simultaneous supralesional sensory-feedback and sublesional motor EES restored both volitional leg movement and evoked proprioceptive percepts, with motor- and sensory-specific parameters individualized by deep-learning methods and participant-directed control (19). These developments recast stimulation parameters as dynamic, intent-contingent variables rather than fixed clinician-programmed constants—a shift with profound implications for how parameter “optimization” should be defined and studied.

#### 3.2.6 Critical appraisal: the evidentiary basis of mechanistic claims

A consistent caveat applies across all five mechanisms above. The cellular and circuit-level evidence—including interneuron identity (28), phenotype switching (36), synergy-based spatiotemporal control (35), and segmental autonomic mapping (38, 39)—derives almost entirely from rodent and non-human primate models, whose dorsal-column geometry, afferent anatomy, and scaling differ materially from the human cord; indeed, proprioceptive cancellation under continuous EES was observed in humans but not rats (31). Human mechanistic evidence remains largely indirect, resting on electrophysiological surrogates such as posterior root–muscle reflexes and evoked EMG responses (32), and on correlational observations in small uncontrolled cohorts; the recent motoneuron-entrainment model (20) is a notable exception in combining human electrophysiology with primate and computational data. Moreover, because clinically complete injuries are frequently discomplete (29) and spared tissue predicts response (30), mechanistic interpretations of “reanimation” must contend with the possibility that EES amplifies residual pathways rather than resurrecting silent ones. Mechanistic claims throughout this review should therefore be read as hypotheses calibrated in animals and only partially verified in humans—a distinction that directly shapes the strength of the parameter recommendations developed in subsequent sections.

## 4 Stimulation parameters and their impact on functional recovery

The efficacy of epidural spinal cord stimulation (EES) in promoting functional recovery after spinal cord injury (SCI) is intrinsically linked to the selection and optimization of its stimulation parameters. Electrode placement, frequency, intensity, pulse width, waveform, and stimulation mode jointly determine how the delivered electrical field interacts with spinal circuitry, and variability in these parameters is a major source of the heterogeneity observed across published outcomes. In this section, we move beyond description: each parameter domain is followed by a critical appraisal of contradictory findings, methodological limitations, and reporting gaps, and each subsection closes with a preliminary observation intended to support protocol design and hypothesis generation, not treatment recommendations. A scenario-based synthesis with descriptive evidence levels is provided in Section 4.7 and Table 1, with levels assigned per the scheme defined in Section 2.3.

### 4.1 Electrode placement and configuration

The anatomical location and configuration of the implanted electrodes are among the most consequential parameters, because they determine which spinal segments and neural structures are recruited. The spinal cord is somatotopically organized, and the EES literature broadly divides into a lumbosacral tradition for lower-limb, trunk, and pelvic-organ functions, and a cervical tradition for upper-limb function.

For lower-limb motor recovery, electrodes are typically implanted over the lumbosacral enlargement (16-contact paddle arrays at the T11–L1 vertebral level, overlying the L1–S1/S2 spinal segments), targeting the segments that innervate the leg muscles and house locomotor central pattern generators (8–10). In individuals with chronic motor-complete paraplegia, lumbosacral EES enabled full weight-bearing standing, with optimal effects when cathodes were placed caudally (10), and similar configurations have been replicated at independent centers (41, 42). For upper-limb recovery, cervical EES targeting the cervical enlargement is required: in non-human primates, lateral cervical electrodes selectively recruited upper-limb motoneurons via sensory afferents in a movement-modulated fashion (37), and spatiotemporal cervical EES restored voluntary arm control in paralyzed monkeys (43). First-in-human work with a 32-contact cervical paddle array has begun to translate this principle to individuals with complete cervical SCI (44) (preprint). The clinical bar for this indication is rising quickly: non-invasive cervical stimulation has already completed a 60-participant pivotal trial (24), so cervical EES will need to demonstrate added value commensurate with its surgical risk. Placement effects also extend beyond the directly innervated segments: lumbosacral, trunk-specific stimulation improved sitting trunk stability in individuals with cervical SCI (45), and preclinical mapping studies show that pelvic-organ targets are also segmentally segregated—detrusor activation with upper- and lower-lumbar stimulation vs. external urethral sphincter activation with sacral stimulation (38, 39)—informing ongoing clinical bladder-targeted programming (46). The number and arrangement of contacts further constrain selectivity: multi-contact arrays (16-contact electrodes; 32-contact paddles) permit flexible spatial programming (44, 47) (both preprints), and circumferential bioelectronics enabling 360-degree recording and stimulation have been demonstrated in preclinical and cadaveric proof-of-concept (48). Mechanistically, these placement and configuration effects follow from how EES recruits neural elements. Delivered over the dorsal cord, EES preferentially activates large myelinated proprioceptive afferents within the posterior roots and dorsal columns (9, 32), so the rostrocaudal lead position determines which root entry zones—and hence which segmental motor pools—are engaged. Because recruitment follows the size principle, with the largest-diameter afferents activated at the lowest thresholds, selectivity is governed jointly by anatomy and electrical configuration: the choice, spacing, and polarity of active contacts (for example, caudally weighted cathodes with rostral anodes steering the current field), together with pulse width, shapes the stimulated volume and the order in which afferent fibers are recruited. This accounts for why nominally identical vertebral placements can yield different recruitment patterns across individuals, and why electrophysiological confirmation of the targeted motor pools at implantation is widely practiced and physiologically motivated (Section 6.3, Step 2).

#### 4.1.1 Critical appraisal

Two systematic biases complicate cross-study comparison of electrode placement. First, reported location is not necessarily actual location. Retrospective MRI reconstruction of implanted leads revealed that leads did not always precisely cover the intended L1–S2 segments, and that minor lead migration—even with stable intraoperative mapping—measurably altered motor evoked potential recruitment curves and muscle coordination (49). Because almost all studies report the intended vertebral target rather than imaging-verified contact positions, placement is a hidden systematic error source whenever protocols are compared across centers. Second, most reports specify vertebral levels, whereas the physiologically relevant variable is the spinal segment covered; the vertebral-to-segment offset varies along the cord and between individuals, further blurring nominally identical placements. Objective electrophysiological aids, such as the peak slope ratio of agonist–antagonist recruitment curves, have been proposed to identify optimal configurations for standing (50), but remain single-center tools.

#### 4.1.2 Preliminary observation

Reported practice converges on selecting the target by functional goal—lumbosacral enlargement (T11–L1 vertebral) for standing/walking and pelvic organs, cervical enlargement for arm/hand function. A further pragmatic observation is that final contact positions should be verified with postoperative imaging rather than operative records alone; where possible, segmental coverage can be confirmed with electrophysiological mapping. These are descriptive observations, not graded recommendations.

### 4.2 Frequency

Stimulation frequency shapes temporal summation of synaptic inputs and network excitability, and the literature reveals a broad functional segregation: low frequencies for motor facilitation, high frequencies for spasticity suppression.

Low-frequency stimulation, typically 25–60 Hz, has been widely used to facilitate standing and volitional movement (10, 47) (the latter a preprint). In motor-complete SCI, standing was optimized at 25–60 Hz with caudal cathode configurations (10), and dual-mode protocols have used 30–60 Hz for the motor-facilitation component (47) (preprint). This range plausibly supports temporal summation of excitatory postsynaptic potentials, bringing motoneurons toward firing threshold. In contrast, high-frequency stimulation in the kilohertz range has emerged for spasticity control: a dual-mode protocol pairing 1.2 kHz stimulation for spasticity with 30–60 Hz for motor facilitation yielded superior and sustained reductions in Modified Ashworth scores compared with rehabilitation alone (47) (preprint), and kilohertz-range high-frequency EES has been reported to abolish patellar hyperreflexia and ankle clonus when paired with low-frequency facilitatory stimulation (51). This newer paradigm contrasts with the historical tonic approach to spasticity, which used intermediate frequencies of 100–130 Hz at thoracolumbar sites (52).

#### 4.2.1 Critical appraisal

Two tensions deserve emphasis. First, the mechanistic explanation for high-frequency spasticity control is inconsistent across sources: some authors attribute the effect to a sustained depolarization (conduction) block of hyperexcitable afferent fibers, whereas others invoke modulation of inhibitory interneuronal circuits that restores reflex gating. These mechanisms make different predictions about optimal frequency, duty cycle, and reversibility, yet no study has tested them head-to-head; the kilohertz values reported clinically are currently empirical, and the exact parameters of the dual-mode paradigm remain incompletely published (47, 51) (the former a preprint). Second, in programmed stimulation the notion of “a frequency” is already obsolete. In the STIMO spatiotemporal protocol, frequency was assigned per motor program: approximately 40–120 Hz favored whole-leg flexion synergies whereas 20–30 Hz favored knee/ankle extensors (12). Frequency thus co-varies with contact selection, gait phase, and task, and quoting a single “optimal frequency” for walking conflates incompatible paradigms. A further caveat: motor-facilitating tonic frequencies can themselves provoke spasms in some individuals, so the facilitatory and anti-spastic frequency regimes are not cleanly separable in practice.

#### 4.2.2 Preliminary observation

For motor facilitation, the frequencies reported across human studies cluster within a 15–60 Hz window, with per-task titration; for spasticity-predominant presentations, a dual-mode strategy (kilohertz-range block plus low-frequency facilitation) has been proposed, but its evidence base is two small case series and one preprint, and its mechanism is unresolved. These observations are hypothesis-generating.

### 4.3 Intensity/amplitude

Intensity determines the spatial extent of neural recruitment and the magnitude of evoked motor responses, and is reported in volts (constant-voltage systems) or milliamperes (constant-current systems).

In practice, intensity is titrated against a physiological target—a motor threshold, a recruitment curve, or a functional behavior. For standing, individual-specific amplitudes (e.g., 1–9 V, constant voltage) proved decisive (10), and the peak slope ratio of agonist–antagonist muscle recruitment curves has been proposed as an objective titration target, with a slope ratio ≥1 associated with better standing performance (50). Intensity must simultaneously respect two constraints. The first is recruitment efficiency vs. tolerance: amplitudes should achieve maximal functional recruitment at the lowest effective and most tolerable level, since discomfort limits therapy duration; related transcutaneous work shows that perceived tolerance tracks muscle-response intensity more than waveform parameters, suggesting that physiological effect, not nominal amplitude, governs acceptability (53). The second is preservation of proprioception: continuous EES at excessive intensities can block proprioceptive input and thereby prevent the online modulation of locomotion (31). Mechanistic work further indicates that the window of “functional” intensities—those within which residual descending input can convert subthreshold stimulation into voluntary motoneuron output—is restricted and narrows with lesion severity (20).

#### 4.3.1 Critical appraisal

There is no consensus titration method. Studies variously set amplitude by motor threshold, by recruitment-curve slope, by evoked potential amplitude, by functional performance, or by patient tolerance, and these anchors are not interchangeable. Worse for evidence synthesis, amplitudes are reported in two non-convertible unit systems: legacy constant-voltage devices report volts, whereas newer constant-current platforms report milliamperes, and across 64 clinical studies amplitudes spanned 0.1–40 V or 0.1–15 mA without any validated crosswalk (16). Because tissue impedance differs across individuals, electrode geometries, and time since implant, volts and milliamperes cannot be retrospectively converted; this unit heterogeneity is a principal technical obstacle to quantitative cross-study synthesis of EES dose.

#### 4.3.2 Preliminary observation

Across the literature, intensity is most interpretable when titrated to an explicit physiological anchor (motor threshold or recruitment-curve slope ratio) rather than to tolerance alone, and reported amplitudes are comparable only when the anchor, the units, and the constant-voltage/constant-current nature of the device are documented.

### 4.4 Pulse width and waveform

Pulse width (PW) and waveform are fundamental determinants of which neural elements are recruited, yet they receive little attention in the SCI literature.

Across 64 clinical eSCS studies, reported PW ranged from 150 to 1,000 μs, with ~450 μs frequently used by the Louisville group and 210 μs by others (8, 16); longer PW recruits more neural elements at a given amplitude but reduces selectivity and increases charge delivery. Waveform conventions differ by platform and era: legacy constant-voltage systems delivered capacitively-decoupled monophasic rectangular pulses with an exponentially decaying charge-balancing phase, whereas newer platforms deliver symmetric or asymmetric biphasic charge-balanced pulses, which minimize net charge and reduce tissue and electrode risk (32, 41). The biophysical basis for these effects is the strength–duration relationship: shorter pulse widths exploit the longer chronaxie of small-diameter fibers to restrict recruitment to large myelinated afferents, improving spatial selectivity and charge efficiency, whereas longer pulse widths lower the amplitude threshold but broaden the recruited fiber population and increase charge injection per pulse. Pulse width therefore trades off against amplitude and contact selection in determining which neural elements are engaged, and cannot be interpreted in isolation from them.

#### 4.4.1 Critical appraisal

PW is frequently unreported even in landmark motor studies—including the papers that anchor the field's claims for standing and walking (10, 11, 31)—and waveform type is almost never justified physiologically. This is not a minor omission: because PW and amplitude trade off in determining charge injection and recruitment, a study reporting amplitude without PW provides an incomplete dose description that cannot be replicated or pooled. We therefore propose that future EES studies adopt a minimum parameter-reporting checklist: (i) imaging-verified electrode position and active-contact configuration; (ii) frequency, specified per program for multi-program paradigms; (iii) amplitude with units, device type (constant voltage vs. constant current), and the physiological anchor used for titration; (iv) pulse width; (v) waveform and charge-balancing scheme; (vi) stimulation mode and duty cycle; and (vii) the titration/optimization protocol itself. Adoption of such a checklist—consistent with recent calls for reporting homogeneity (14, 16)—would materially improve reproducibility and enable quantitative synthesis.

#### 4.4.2 Preliminary observation

Reports lacking PW (or amplitude units) provide an incomplete dose description and cannot be interpreted or replicated quantitatively; typical contemporary practice uses biphasic charge-balanced pulses of 210–500 μs, but no evidence favors one value within this range for a given indication.

### 4.5 Stimulation modes: tonic, burst, spatiotemporal, and dual-mode

Beyond individual parameters, the temporal patterning of delivery—the stimulation mode—critically shapes outcomes, and it is here that the field's most instructive contradiction resides.

Tonic (continuous, constant-frequency) stimulation provides a stable facilitatory background and underpinned the field's foundational demonstrations: full weight-bearing standing in motor-complete SCI (10), over-ground walking after intensive training (11), and volitional leg movement in 13/13 motor-complete participants (30). Burst and spatiotemporal stimulation were developed to mimic physiological activation patterns and to preserve proprioceptive feedback: delivering pulses in bursts, or sequencing contacts in time with the gait cycle, mitigated the proprioceptive cancellation seen with continuous EES and enabled adaptive locomotion in humans (12, 31); in rats, synergy-timed spatiotemporal neuromodulation outperformed continuous stimulation (35). Spatiotemporal EES combined with neurorehabilitation restored walking in nine participants, including some with complete sensorimotor paralysis (28). Dual-mode stimulation deliberately combines parameter sets—kilohertz-range stimulation for spasticity with low-frequency stimulation for motor facilitation—to treat multiple problems with one implant (47, 51) (the former a preprint).

#### 4.5.1 Critical appraisal

The central contradiction: Formento et al. (31) showed, with convergent computational, preclinical, and human evidence, that continuous EES blocks proprioceptive input in humans (“transient deafferentation”) and concluded that stimulation must preserve proprioception to enable locomotion—yet tonic continuous stimulation produced the field's most-cited functional gains in the Louisville cohorts, including standing and over-ground walking (10, 11, 30). Several factors plausibly reconcile the discrepancy. (i) Task type: proprioceptive feedback is most critical for dynamic, adaptive locomotion over uneven terrain, whereas static standing and stereotyped treadmill stepping can be sustained by a tonic facilitatory set-point with less dependence on online feedback. (ii) Cohort composition: the proprioception-blocking demonstrations and the spatiotemporal superiority data derive largely from motor-incomplete (AIS C/D) participants with partially preserved sensory channels, whereas the tonic successes were in AIS A/B participants in whom little proprioceptive transmission remains to be disrupted—continuous stimulation may carry no sensory cost when the sensory pathway is already interrupted. (iii) Field geometry: wide-field single-configuration tonic stimulation and multi-contact timed sequences activate afferents in fundamentally different spatiotemporal patterns, so “continuous vs. patterned” comparisons conflate mode with spatial selectivity. Declared device interests on both sides of this debate additionally warrant caution when interpreting paradigm-superiority claims. A balanced interpretation is that the mode–proprioception trade-off is real but task- and individual-dependent, not absolute.

#### 4.5.2 Preliminary observation

Reported programs suggest a tentative pattern of mode–task matching: tonic configurations have been used most often for standing and for motor-complete presentations, whereas spatiotemporal (and, where available, burst) programs have been applied mainly to dynamic walking in individuals with preserved proprioception; dual-mode strategies for combined spasticity–weakness presentations remain early-phase. Controlled comparisons of modes against a matched co-training background are lacking.

### 4.6 Open-loop vs. closed-loop systems

Most clinical EES remains open-loop: parameters are pre-programmed or manually adjusted by clinicians and do not adapt in real time to the user's state or intent. Closed-loop systems integrate physiological or neural feedback to modulate stimulation dynamically and represent the field's leading edge.

Preclinical milestones established feasibility: a brain–spine interface decoding leg motor-cortex activity to trigger EES restored weight-bearing locomotion in non-human primates (18); local-field-potential-driven cervical stimulation improved forelimb function in rats (40); and closed-loop epidural stimulation elicited respiratory plasticity after acute SCI in rodents (54). Translation to humans has accelerated markedly. A fully implanted brain–spine “digital bridge” linking cortical decoding to analog modulation of lumbosacral EES restored natural standing, walking, and stair climbing in a participant with chronic incomplete tetraplegia, with stable calibration over more than a year of home use and neurological recovery persisting with the device off (17). Perilesional bidirectional stimulation—simultaneous supralesional sensory-feedback EES and sublesional motor EES, with parameters individualized by deep-learning methods and participant-directed control—restored volitional leg movement together with evoked proprioceptive feedback in three individuals with motor-complete SCI (19). Closed-loop control has also reached autonomic indications: a biomimetic “neuroprosthetic baroreflex” prevented orthostatic hypotension across rats, non-human primates, and a human participant (55), and proportional-integral-derivative closed-loop hemodynamic stimulation produced durable blood-pressure stability in a 14-participant series (56). AI-driven parameter optimization and computational modeling are expected to accelerate this transition (20, 57).

#### 4.6.1 Critical appraisal

The closed-loop evidence is promising but preliminary: the landmark human demonstrations are *n* = 1–3, device-specific, and inseparable from months of co-delivered rehabilitation; durability, failure modes of decoders, and cost-effectiveness are unknown. Open-loop tonic stimulation, by contrast, has been replicated across centers for over a decade. Closed-loop systems should presently be viewed as a research trajectory with strong proof-of-concept rather than a clinically available standard.

#### 4.6.2 Preliminary observation

Open-loop stimulation remains the most extensively reported configuration; closed-loop and brain–spine approaches are emerging and, at present, most appropriately accessed through clinical trials, particularly for patients with sufficient cortical signal and rehabilitation access.

### 4.7 Synthesis: parameter selection by clinical scenario

Integrating the evidence above, two broad parameter philosophies recur across indications. The first—the tonic, individually titrated philosophy—uses a lumbosacral (or cervical) paddle, a single wide-field configuration, low-to-moderate frequencies (15–60 Hz), amplitudes anchored to motor threshold, and relies on months of co-delivered activity-based training; it accounts for most standing, volitional-movement, bladder, and early cardiovascular results. The second—the programmed, biomimetic philosophy—distributes stimulation across contacts, frequencies, and time (spatiotemporal, dual-mode, or closed-loop), assigns parameters per motor program or per physiological feedback signal, and dominates recent walking and hemodynamic-control advances.

The maturity of parameter evidence differs sharply by scenario (Table 1). Standing and walking are supported by converging parameter ranges from multiple independent centers, although every contributing study is a case report or uncontrolled series. Blood-pressure management now has the most standardized parameter set of any indication—a lower-thoracic hemodynamic hotspot (the last three thoracic segments, explicitly shown to outperform lumbosacral targeting), 120 Hz, ~300 μs, and amplitudes titrated to a pressor response under continuous or closed-loop control (55, 56)—superseding earlier lumbosacral, individually optimized approaches (58). Upper-limb cervical EES is mechanistically strong but clinically early-stage, resting on one two-participant human study and non-human primate work, though systematic frequency–motor-pool mapping is beginning to appear (37, 44) (the latter a preprint). For spasticity, the historical 100–130 Hz tonic paradigm (52) and the newer kilohertz-block dual-mode paradigm (47, 51) (the former a preprint) coexist without any dose-finding trial to adjudicate between them. Bladder outcomes are derived almost entirely from one center's mapping programs, and bowel benefits remain secondary, self-reported endpoints without a dedicated parameter set. Finally, for neuropathic pain after SCI there is no SCI-specific parameter evidence at all: paradigms are adopted directly from conventional pain practice (tonic 30–80 Hz; burst; 1–10 kHz high-frequency), a systematic review graded the entire evidence base as low quality with no RCTs (59), and the only modern case series of burst or 1.2 kHz stimulation in SCI-related pain reported satisfactory relief in just two of eight patients—both with incomplete injuries (60).

Three caveats govern the use of Table 1. First, parameters in this literature are individually optimized within single sessions or series and have almost never been compared in dose–response designs; the table therefore presents ranges and target physiology, not prescriptions. Second, constant-voltage (V) and constant-current (mA) amplitude systems are not directly comparable, and values should be interpreted only within their original unit system. Third, evidence levels are stated explicitly for each scenario and should temper clinical confidence accordingly.

## 5 Clinical outcomes and modifiers of recovery

Epidural spinal cord stimulation (EES) has been associated with functional gains across motor, sensory, and autonomic domains, and with relief of secondary complications such as spasticity and pain. These outcomes, however, must be interpreted against the design limitations of the underlying literature—predominantly uncontrolled case reports and case series with intensive rehabilitation co-interventions. Accordingly, each subsection below closes with an explicit evidence-level appraisal, and Section 5.4 directly addresses the contradictions and methodological heterogeneity that complicate any parameter-level synthesis. Because stimulation was almost always delivered together with intensive, task-specific rehabilitation, the outcomes summarized below are those of combined stimulation-plus-training interventions (Section 5.4e).

### 5.1 Motor outcomes

#### 5.1.1 Standing

Standing is the most replicated EES outcome, and the concurrent training background is comparatively well-documented. Following the single-case demonstration that lumbosacral EES enabled full weight-bearing standing after motor-complete paraplegia—in a participant who had completed 170 pre-implant locomotor-training sessions over 26 months and then 80 post-implant stand-training sessions (60 min each) over approximately 7 months (8)—Rejc et al. (10) showed that individually optimized tonic stimulation (25–60 Hz, caudally weighted cathodes) enabled independent standing in 4/4 motor-complete participants undergoing stand training at 1 h per session, 5 sessions per week (~80 sessions), and volitional leg movement was subsequently elicited in 13/13 motor-complete participants—in whom the ability to generate force or movement did not correlate with any neuroimaging marker, whereas select EMG coordination features correlated with spared spinal tissue on MRI (30). Independent cohorts have since reproduced EES-enabled standing: in a cervical-SCI cohort undergoing stand-focused activity-based recovery training (stand-ABRT, 2 h/day, 5 days/week; 80–160 sessions depending on group) plus scES, 97% of participants achieved 2 h of standing by the end of the intervention, with the 2-h goal reached in ~70% of training sessions (33); 3/3 participants with sensorimotor complete (AIS A) thoracic SCI achieved independent standing with a 32-contact paddle, supported by home-based stimulation use of ~2–3 h/day over approximately 12 months rather than a supervised clinic training protocol (65); and 4/5 previously non-standing participants stood full weight-bearing in an Indian pilot cohort, in which the locomotor training delivered with stimulation was not reported in detail (42). Evidence appraisal: standing is supported by convergent results from at least three independent centers, but the evidence consists almost entirely of uncontrolled case reports and case series with *n* ≤ 10 per cohort; effects are largely stimulation-dependent (“prosthetic”), and were achieved together with concurrent task-specific stand training as components of a combined intervention (Section 5.4e). Stimulation sets the excitability state of the relevant networks, while training provides the functional context in which standing is relearned.

#### 5.1.2 Walking/locomotion

Two parameter philosophies have produced walking after SCI, and in both, stimulation was embedded in structured locomotor training of widely differing dose. Continuous, task-specific tonic configurations combined with intensive ABRT restored over-ground walking in 2/4 motor-complete participants—both AIS B (sensory-spared)—only after 81–278 training sessions (1 h each, once or twice daily) over 15–85 weeks (11); Gill et al. (41) reported independent treadmill stepping after complete paraplegia with a similar tonic approach combined with 43 weeks of multimodal rehabilitation (~3 sessions/week; standing, treadmill and overground stepping). Spatiotemporal multi-contact programs—an approach originating in rodent work showing that synergy-timed stimulation outperforms continuous stimulation (35)—restored adaptive walking within 1 week in three motor-incomplete participants (12) and walking capacity in nine participants including some with complete sensorimotor paralysis (28, 61); in all three studies, stimulation was paired with a standardized neurorehabilitation program (4–5 sessions/week, ~2 h per session, for ~5 months, combining body-weight-supported treadmill and overground walking with robotic assistance that was progressively withdrawn); a cortically driven brain–spine interface later enabled natural over-ground walking in a single participant within the same rehabilitation framework (17). More recently, EES combined with a multimodal program—motor imagery (clinic and home practice), trunk rehabilitation, and robotic-exoskeleton overground gait training (5 sessions/week, 1–1.5 h, for 7 months; EksoGT 2–3 × /week, 1,000–1,800 steps per 45–60-min session)—produced independent over-ground walking in two motor-complete participants within 1 month of the training period (66), and the first controlled (non-randomized) cohort showed greater improvements in lower-extremity motor scores and walking with EES plus physical therapy (cycling, strengthening, continuous passive motion, and body-weight-supported treadmill training, 4–5 h/day with EES continuously on; weekly frequency and total sessions not reported in detail) than with physical therapy alone (67) (*n* = 21). Gains on the Walking Index for Spinal Cord Injury II (WISCI-II) sustained over 24 months have also been reported (68). Hybrid 12-month exoskeleton training with percutaneous epidural stimulation has likewise been described (91), and both a health-technology assessment (92) and a recent review (93) of robot-assisted rehabilitation combined with spinal neuromodulation have appeared. Evidence appraisal: the largest controlled EES cohort to date (67) (*n* = 21) is non-randomized; all landmark studies enrolled *n* = 1–9 in single-arm designs, and no sham-controlled RCT of EES for walking exists. Retained (stimulator-off) improvements have been reported, and their magnitude varies with the dose and protocol of the accompanying training, which differed widely across studies (Section 5.4e). Across these studies, stimulation was embedded in task-specific locomotor practice—body-weight-supported treadmill training, over-ground training, robotic assistance, or mental imagery—and this coupling is essential for interpretation, particularly for readers outside the field: stimulation alone may evoke non-coordinated muscle contractions, but coordinated, functional locomotion emerges only when stimulation is paired with task-specific training.

#### 5.1.3 Upper-limb function

Cervical EES for arm and hand recovery remains early-stage. Mechanistic work in non-human primates established that cervical dorsal-root stimulation recruits upper-limb motoneurons via sensory afferents (37) and can restore voluntary upper-limb control after cervical SCI (43). In humans, a 32-contact cervical paddle with systematic location-, intensity-, and frequency-based mapping, combined with goal-directed hand-task practice (reach–grasp–lift–release activities) and continued autonomous home and community use of personalized programs over several months, restored elements of volitional hand open/close and reach–grasp function in two participants with AIS A tetraplegia (44) (preprint; structured session frequency and duration not reported in the accessible report). Mechanistically, the “functional” parameter space in which residual descending input can be converted into volitional motoneuron output has been shown to narrow as lesion severity increases (20). The only large pivotal trial in this space is transcutaneous, not epidural: the single-arm Up-LIFT trial (*n* = 60 completers) met its endpoints and led to FDA *De Novo* authorization of the ARC-EX system (24). Evidence appraisal: human cervical EES evidence rests on a single two-participant preprint; the largest dataset is a single-arm transcutaneous trial—efficacy claims for epidural upper-limb stimulation rest on preclinical coherence rather than controlled human evidence. Stimulation in these studies was likewise combined with task-specific upper-limb practice (reach-and-grasp and hand-opening exercises); as in locomotion, stimulation-evoked contraction should not be conflated with functional arm and hand movement, which develops through training under stimulation.

#### 5.1.4 Trunk stability and postural control

Trunk-specific lumbosacral scES improved sitting trunk lean distances and trunk-muscle EMG activity in individuals with severe cervical SCI in an acute assessment study that delivered no concurrent training—participants had no prior intervention sessions with trunk-specific scES—indicating that these immediate effects reflect stimulation-evoked postural facilitation rather than trained skill (45). Activity-dependent programs rapidly restored trunk control after complete paralysis within the STIMO rehabilitation framework (4–5 sessions/week for ~5 months) (61). However, robotic postural stand training (RobUST; 1 h/day, 5 days/week; 80 ± 10 sessions over ~5.2 ± 1.1 months) with stand-targeted scES did not improve sitting postural control (69), indicating that postural gains are task- and program-specific rather than generalized (Section 5.4b). Evidence appraisal: two small studies with directly conflicting conclusions; insufficient to support any trunk-specific parameter recommendation. These studies also illustrate the distinction between acute stimulation-evoked facilitation of trunk musculature (45) and trained functional postural control (69), and reinforce that functional gains track the trained task.

### 5.2 Sensory and autonomic outcomes

#### 5.2.1 Sensory function

Significant sensory improvements were observed in the controlled cohort of Ren et al. (67) (EES + physical therapy vs. physical therapy alone), and increased ASIA sensory scores accompanied dual-mode stimulation in a recent preprint (47). In sensorimotor complete SCI, sensory gains cluster within approximately three segments of the neurological level of injury and are substantially larger in sensory-incomplete injuries (~30 vs. ~6% gains in complete injuries) (70). Evidence appraisal: sensory outcomes are almost invariably secondary endpoints in uncontrolled studies; consistent in direction but sparse in measurement detail.

#### 5.2.2 Blood pressure and cardiovascular regulation

This is the most mature and parameter-standardized autonomic indication. Lumbosacral scES configured for motor tasks acutely raised arterial pressure during orthostatic challenge in hypotensive individuals [119/72 vs. 70/45 mmHg with vs. without stimulation; (58)], and a biomimetic closed-loop “neuroprosthetic baroreflex” prevented orthostatic hypotension across rats, non-human primates, and one human participant (55). A recent multi-trial series (*n* = 14) refined the paradigm substantially: targeting the lower-thoracic hemodynamic “hotspot” (the last three thoracic segments, shown head-to-head to outperform lumbosacral placement) with 120 Hz, ~300 μs pulse width, and amplitudes titrated to the pressor response—under continuous or PID closed-loop control—produced durable hemodynamic stability and discontinuation of pressor medications (56). Conventionally positioned lumbosacral arrays can also be re-parameterized to blunt autonomic dysreflexia (22) (*n* = 3), and a 2026 systematic review confirms consistent acute pressor effects while flagging frequency-dependent risks (e.g., exacerbation of autonomic dysreflexia at high tSCS frequencies) (23). Ergogenic cardiovascular benefits during exercise have been reported with individually mapped configurations (71). Evidence appraisal: convergent, mechanism-anchored, and now standardized (hotspot level, 120 Hz, closed-loop titration), but still based on small uncontrolled series totaling fewer than 20 participants; no controlled trial exists.

#### 5.2.3 Bladder, bowel, and sexual function

In the only center with bladder-targeted programs, urodynamic testing identified ~30 Hz as optimal for voiding efficiency and post-void residual (62); motor-configured scES with ABRT increased bladder capacity and lowered detrusor pressure, although bladder-distention–evoked blood pressure elevation persisted, arguing for intersystem (bladder + cardiovascular) programming (13); and network-specific bladder mapping programs further improved capacity and detrusor pressure in a participant-specific manner (63) (*n* = 7). Independent corroboration includes improved urodynamics, neurogenic bowel scores, and erectile function under chronic EES (65) and immediate autonomic pathway facilitation including restored orgasm in one participant (7) (*n* = 2). Preclinical mapping provides a segmental rationale [detrusor at L1/L5–L6, external urethral sphincter at sacral levels; (38, 39)], and a 2026 scoping review of 68 studies confirms autonomic benefits while emphasizing case-report dominance (21). Evidence appraisal: consistent but uncontrolled, largely single-center (Louisville) case series with *n* ≤ 10; bowel outcomes are secondary and self-reported, and no bowel-specific parameter set has been developed.

### 5.3 Spasticity and pain

#### 5.3.1 Spasticity

Historical series used tonic lumbar stimulation at 50–100 Hz (72) (*n* = 8) or 100–130 Hz (52) (*n* = 19 with spinal spasticity), reporting meaningful Ashworth reductions. A systematic review pooling 34 studies found a 78% rate of subjective improvement but graded the literature as low-quality and highly heterogeneous (73). A mechanistically distinct paradigm has now emerged: kilohertz-range high-frequency EES acts as a reversible conduction block that abolished patellar hyperreflexia and ankle clonus, while pairing with low-frequency facilitatory EES improved walking (51) (*n* = 2); a dual-mode protocol (1.2 kHz for spasticity + 30–60 Hz for motor facilitation) similarly reported sustained Modified Ashworth Scale reduction (47) (preprint). Transcutaneous studies show only temporary attenuation (74). A practical tension should be noted: motor-facilitating tonic frequencies can themselves provoke spasms in some patients, and no dose-finding trial exists for either paradigm. Evidence appraisal: small, old, uncontrolled series plus a two-participant new paradigm; low overall quality with no comparative parameter data.

#### 5.3.2 Neuropathic pain

This is the weakest outcome domain. A systematic review of 69 SCS-treated patients with SCI neuropathic pain rated the evidence as low quality on the Grading of Recommendations Assessment, Development and Evaluation (GRADE) scale and identified no RCT (59). Modern paradigms fare little better: with burst or 1.2-kHz high-frequency SCS, only 2/8 participants (both incomplete SCI) reported satisfactory relief at 12 months (60). Positive signals exist only as secondary outcomes within motor/autonomic studies (47, 67) (the former a preprint). Stimulation parameters are adopted directly from chronic-pain practice (tonic 30–80 Hz, HF 1–10 kHz, burst 40/500 Hz) with no SCI-specific optimization, and efficacy is poorest for below-level pain after complete injury, plausibly reflecting spinothalamic-tract interruption. Evidence appraisal: GRADE-low; current evidence does not support parameter recommendations for pain in SCI, and this should be stated plainly to patients.

### 5.4 Contradictions and methodological heterogeneity across studies

(a) Controlled trials vs. the uncontrolled literature. The field's most rigorous test to date—the eWALK trial (*n* = 50, seven sites), a triple-blind, sham-controlled RCT of tSCS plus locomotor training—found no between-group difference on any outcome (primary: −0.1 WISCI-II levels, 95% confidence interval −1.6 to 1.5, *p* = 0.98), while both arms improved ~2.5 WISCI-II levels from training alone (75) (protocol: (76)). Because eWALK tested transcutaneous rather than epidural stimulation, this null result constrains the non-invasive neuromodulation principle and constitutes only indirect evidence regarding epidural protocols (Section 5.4c). This null result stands in sharp contrast to near-universally positive uncontrolled reports: a dedicated scoping review of epidural SCS found that every included publication was supportive, “with no reports of significant failures” (15). The one positive RCT of tSCS (77) (*n* = 27) lacked an *a priori* power calculation and enrolled subacute participants, in whom spontaneous recovery confounds attribution. Critically, no sham-controlled RCT of epidural stimulation for motor recovery has been published; the field's only regulatory approval (ARC-EX; FDA *De Novo*, December 2024) rests on the single-arm, open-label Up-LIFT trial (24). Placebo-mediated neurological improvement is documented in SCI (78, 79), sham surgery is ethically and practically impossible (80), and perceptible stimulation makes participant blinding structurally difficult (81). The most defensible reading is twofold: intensive locomotor training is itself a potent active ingredient of combined interventions, and a stimulation-specific increment of transcutaneous stimulation over high-dose training was not detectable at this sample size. Extrapolation to epidural protocols—which afford far greater spatial selectivity and more standardized dosing (Section 5.4c)—is unwarranted; epidural evidence must ultimately be judged on controlled epidural trials.
(b) Task specificity: standing vs. sitting. Robotic postural stand training with scES improved standing but produced no transfer to sitting postural control (69), whereas trunk-specific scES explicitly programmed for sitting improved sitting trunk stability (45). Read together, these studies are less contradictory than instructive: EES gains reflect task-specific motor learning tied to the trained posture and the activated program, not generalized postural recovery. Parameter libraries must therefore be scenario-specific, and success in one task cannot be extrapolated to another.
(c) EES vs. tSCS: equivalence or fundamental difference? Epidural and transcutaneous lumbar stimulation elicit posterior root–muscle reflexes through common neural structures, supporting claims of physiological equivalence and a preference for the non-invasive modality (32). However, equivalence of acute reflex circuitry is not equivalence of therapeutic efficacy: fundamental physiological differences between the two modalities have been emphasized (82), tSCS lacks the spatial selectivity and standardized dosing of epidural arrays (83), the modalities carry different risk profiles (surgical complications and cardiovascular events under EES; (84, 85)), and no randomized head-to-head comparison exists. Claims of interchangeability—on which some cross-modality evidence borrowing rests—should be treated as unproven. Accordingly, throughout this review, transcutaneous evidence—including eWALK (75), Up-LIFT (24), and Comino-Suárez et al. (77)—is cited only to inform questions of evidence hierarchy, trial design, and clinical translation; all efficacy and parameter conclusions concerning epidural stimulation are drawn from epidural studies, and wherever transcutaneous findings are discussed alongside them, the modality distinction is flagged explicitly.
(d) Recovery in “complete” SCI: reanimation or unmasking? Voluntary movement under EES in AIS A participants has been interpreted as reactivation of dormant networks (7, 86). The discomplete-SCI construct, however, established three decades ago that clinically complete injuries often retain subclinical brain influence (29), and the EES data are consistent with a residual-pathway requirement: in the NEJM series, only the two AIS B (sensory-spared) participants achieved over-ground walking while both AIS A participants did not (11), and MRI-documented spared tissue predicted volitional responses (30). Mechanistically, EES appears to convert subthreshold residual descending drive into suprathreshold motoneuron output, with the effective parameter window narrowing as lesion severity increases (20). The implication for parameter science is profound: reported “optimal parameters” may index residual anatomy rather than any universal dose–response, arguing for imaging- and electrophysiology-based stratification in all future parameter studies.
(e) Stimulation and training: a combined intervention, not a confound. Virtually every positive EES study delivers stimulation together with intensive, task-specific training-−81–278 sessions in Angeli et al. (11) and structured activity-based recovery training in Angeli et al. (33) and Herrity et al. (13)—and this coupling is inherent to the therapy rather than a methodological flaw. After SCI, sublesional networks persist in a new state in which function must be relearned through the building and consolidation of connections; stimulation changes the excitability state of these networks but does not by itself provide the task-specific information needed to organize circuits for function (Section 3.2.1). Training supplies precisely that information. Training is therefore not a factor to be statistically isolated from stimulation but the functional context in which stimulation becomes therapeutic—much as a plasticity-enhancing drug can only be meaningfully evaluated in learners actually attending language classes: the treatment effect is defined within the learning task, and demanding evidence from “stimulation without training” misconstrues how motor function is restored. Consistent with this framing, evidence throughout this review is presented as evidence for combined stimulation-plus-training interventions, and gains should be attributed to the combination. What remains genuinely unresolved is not whether training “confounds” stimulation but the wide variability of training protocols and doses across studies—from ~80 sessions (Harkema et al. (8); Rejc et al. (10); Angeli et al. (33) low-dose groups) to 160 sessions (Louisville ABRT-scES cohorts (13); Angeli et al. (33) high-dose groups) and up to 278 sessions over 85 weeks (11), with weekly frequency clustering at 4–5 sessions but session durations spanning 1 h to 4–5 h/day (67), and modalities spanning weight-bearing stand training, body-weight-supported treadmill and over-ground stepping, robotic postural and exoskeleton training (66, 69), seated cardiovascular training, motor imagery, and goal-directed hand-task practice (44)—and the resulting uncertainty about minimally sufficient training dose and the dose–response relationship between training intensity and stimulation-enabled gains; eWALK's ~2.5-level WISCI-II improvement in both arms demonstrates that high-quality locomotor training is itself a potent active ingredient of the combined intervention (75). Standardized reporting of training type and dose, and dose-finding designs for the training component, are therefore methodological priorities on a par with standardized parameter reporting.

### 5.5 Modifiers of recovery

Injury completeness is the strongest modifier. Incomplete SCI shows larger and more consistent gains (67, 87), with sensory recovery roughly fivefold greater in sensory-incomplete than sensory-complete injuries (70). Yet meaningful stimulation-dependent function—standing, volitional movement, autonomic gains—remains achievable in AIS A/B injuries (7, 8, 10, 65), likely reflecting residual discomplete pathways rather than *de novo* network reanimation (Section 5.4d).

Injury level dictates targeting: cervical enlargement for upper-limb function (37, 44) (the latter a preprint), lumbosacral segments for standing, walking, and trunk control (10, 45), and the lower-thoracic hotspot for blood pressure (56). Multisite stimulation may benefit high-level injuries, although this observation derives from a single transcutaneous (tSCS) single-subject study (88) (transcutaneous; contextual evidence only).

#### 5.5.1 Chronicity

Most evidence derives from chronic SCI, where durable gains over 24 months are documented (68). Subacute intervention is feasible, including with transcutaneous approaches, (89) but interpretation is confounded by spontaneous recovery—an important caveat when comparing positive subacute trials (77) with null chronic trials.

Lesion characteristics refine prognosis: shorter lesion length and a preserved tissue bridge predict EES-enabled standing (90), and spared tissue on MRI predicts volitional responses (30). These markers should move patient selection from clinical labels toward anatomy- and physiology-based stratification.

#### 5.5.2 Combined therapies

The most robust outcomes occur when EES is embedded in multimodal rehabilitation: task-specific physical therapy/ABRT (33, 67, 68), robotic or exoskeleton assistance (66, 91–93), brain–spine interfaces for intent-driven control (17, 18), and bidirectional perilesional stimulation with deep-learning–optimized parameters (19). Preclinical combinations with cell-based or gene therapies show synergy (94, 95). Two caveats temper enthusiasm: benefits remain task-specific (69), and co-interventions differ widely in protocol and dose across studies; standardized reporting of training type and dose alongside stimulation parameters, together with dose-finding designs for the training component, is a methodological priority (Section 5.4e).

## 6 Critical appraisal, challenges and future directions

### 6.1 State of the evidence

The evidentiary foundations of EES for SCI are weaker than the headline results suggest. A scoping review of all human SCI–SCS research up to 2021 identified 103 studies (55 epidural, 36 transcutaneous, 12 magnetic) containing exactly one randomized controlled trial; 70% enrolled 10 or fewer participants; stimulation parameters were incompletely described across many studies; only 18/103 studies documented any adverse event; and no study reported patient or consumer engagement (14). A focused review of the epidural motor literature could aggregate only 13 case series totaling 88 participants and could not perform any formal quality or risk-of-bias assessment—while noting that every included publication was supportive, an asymmetry diagnostic of reporting and publication bias (15). The most recent systematic synthesis (64 studies, 306 patients) documents the same heterogeneity at the parameter level: frequencies spanning 0.2–400 Hz, pulse widths 150–1,000 μs, and amplitudes reported in incompatible units (0.1–40 V constant-voltage vs. 0.1–15 mA constant-current), with T11–L1 the most common lead site (16).

Against this backdrop, two facts define the current evidence hierarchy. First, when the neuromodulation principle was finally tested under randomized, triple-blind, sham-controlled conditions, the added effect over training was null [eWALK, *n* = 50, transcutaneous stimulation—only indirect evidence for epidural protocols, Section 5.4; (75)], while the single positive RCT was small, unpowered *a priori*, and enrolled subacute participants susceptible to spontaneous-recovery confounding (77). Second, a regulatory asymmetry has emerged: the field's only cleared device (ARC-EX, transcutaneous) was authorized on the basis of a single-arm, open-label trial (24), meaning that commercial translation and evidence level are currently decoupled. Blinding and sham design remain structurally unresolved for spinal stimulation (80, 81), and safety ascertainment lags efficacy reporting—dedicated analysis documents post-surgical complications and cardiovascular events (84), and training-related injuries (hip fracture; (11)) and stimulation-related autonomic dysreflexia and skin burns (75) have all been reported (Section 6.2). Parameter-level conclusions in this review, including those summarized in Tables 1, 2, should therefore be interpreted as maps of practice and hypotheses for testing, not as evidence-based prescriptions.

### 6.2 Adverse events and practical risks

Systematic safety reporting is the exception in this literature—only 18 of 103 human studies documented any adverse event (14)—but the available data delineate several risk domains. Surgical and hardware-related complications of epidural implantation include wound infection, lead fracture or migration, cerebrospinal fluid leak, and revision surgery: a dedicated analysis of implanted cohorts documented post-surgical complications alongside cardiovascular events (84), and experience with earlier epidural systems motivated the shift toward percutaneous leads (85). Where systematic surveillance has been performed, results are more reassuring: in the E-STAND pilot (*n* = 14, motor-complete SCI), no surgical complications occurred (0/14), one device malfunction required attention at 4 months (1/14), and stimulation used for up to 23 h/day produced no stimulation-related adverse events, with tilt-table testing showing no significant systolic blood-pressure increases and no arrhythmias (84); in an Indian pilot cohort, no intraoperative or early postoperative complications occurred, although 2/5 participants developed wound complications at 1 and 3 months that were managed successfully (42). Even absent frank complications, lead position is physiologically unstable: retrospective MRI reconstruction showed that minor lead migration measurably altered motor evoked potential recruitment curves and muscle coordination (49), so both efficacy and tolerability can drift over time and may require periodic re-mapping.

Stimulation-related physiological risks include the provocation of spasms at motor-facilitating frequencies, discomfort that limits therapy dose, and autonomic dysreflexia: stimulation-related autonomic dysreflexia and skin burns were reported in the transcutaneous eWALK trial (75), and high-frequency transcutaneous stimulation can exacerbate autonomic dysreflexia (23), whereas appropriately parameterized lumbosacral EES has conversely been shown to blunt it (22). Rehabilitation itself carries risk, with training-related injuries including hip fracture reported during intensive locomotor training under stimulation (11). Finally, because nearly all reported improvements were achieved with concurrent intensive rehabilitation, they reflect the combined intervention and cannot be attributed to stimulation alone; EES is therefore best communicated to patients as an enabling component of a combined stimulation-plus-training program, and structured surveillance of surgical, cardiovascular, stimulation-related, and training-related events should be routine in both trials and practice (Section 6.3, Step 5).

### 6.3 Clinical translation pathway: a decision-oriented framework

Pending controlled trials, any clinical use of EES rests on incomplete evidence. We therefore offer the following five-step pathway as a framework for structured, protocolized practice and research—converting the mapped parameter evidence into a reproducible workflow—rather than as practice-defining guidance.

Step 1—Patient selection and stratification. Beyond the International Standards for Neurological Classification of SCI (ISNCSCI) classification, pre-implant MRI should assess lesion length and the presence of a tissue bridge, both of which predict EES-enabled standing (90), and spared tissue, which predicts volitional responses (30). The discomplete-SCI construct (29) and mechanistic evidence that the functional stimulation window narrows with lesion severity (20) imply that electrophysiological assessment of residual descending drive should complement imaging. Candidates should be counseled honestly that benefits are probable for standing and blood-pressure support, possible for walking and bladder function, and uncertain for pain—graded according to Table 2.

Step 2—Implantation and intraoperative mapping. Evoked-response mapping should confirm recruitment of target motor pools, recognizing that retrospective MRI reconstruction reveals lead migration and suboptimal segmental coverage that degrade recruitment curves (49).

Step 3—Physiology-anchored parameter titration. Amplitude should be titrated against recruitment curves rather than comfort alone; the agonist–antagonist peak slope ratio (≥1) offers an objective criterion for standing configurations (50). For locomotor goals, parameter windows must preserve proprioceptive feedback (31).

Step 4—Scenario-based program libraries. Initial programs may be selected by clinical scenario from the parameter ranges synthesized in Table 1 (preliminary, hypothesis-generating ranges; Section 2.3; e.g., tonic lumbosacral 15–60 Hz for standing; spatiotemporal programs for walking; lower-thoracic hotspot at 120 Hz for blood pressure; kHz-range block paired with low-frequency facilitation for spasticity), then individualized. Because gains are task- and program-specific (45, 69), each functional goal requires its own tested program rather than extrapolation from another. The hemodynamic indication—where a defined hotspot, frequency, and closed-loop titration have been convergently established (55, 56)—provides the template toward which other indications should evolve.

Step 5—Long-term follow-up and safety surveillance. Durable benefits over 24 months are documented (68), but systematic adverse-event reporting is the exception (18/103 studies; (14)); structured surveillance of surgical, cardiovascular, stimulation-related, and training-related events (84) (Section 6.2) should be routine.

#### 6.3.1 Cost and accessibility

EES requires surgical expertise and costly implanted hardware, limiting access particularly in resource-constrained systems (97). Home-deployable transcutaneous systems (98) may serve as a lower-cost triage tier that identifies responders before implantation, but formal cost-effectiveness analyses are absent and needed before widespread adoption can be justified.

### 6.4 Future directions

Progress now depends on closing the loop—technologically and methodologically. Closed-loop, intent-driven systems are the clearest technical trajectory: cortically decoded brain–spine interfaces (17), bidirectional perilesional stimulation with deep-learning–optimized and participant-directed parameters (19), and AI-assisted parameter selection and outcome prediction (57, 99, 100) promise to replace empirical trial-and-error titration. Hardware innovation—circumferential 360-degree electrode arrays (48) and self-powered implants (101)—may improve selectivity and reduce revision burden. Biomarker- and imaging-based stratification (tissue bridge, spared-tissue metrics; (30, 90)) should become standard entry criteria, and multimodal combinations [robotics, cell and gene therapies; (94, 95)] merit controlled testing rather than additive case reports. Methodologically, three priorities dominate: (i) a standardized parameter-reporting checklist—electrode configuration and level, frequency, amplitude with units, pulse width, waveform, duty cycle, and concurrent training dose—addressing the reporting gaps documented repeatedly (14, 15, 83); (ii) adequately powered, sham-controlled RCTs of epidural stimulation with allocation concealment, pre-registered responder definitions, and controlled training dose (15, 80); and (iii) prospective long-term safety registries. Until such trials exist, parameter recommendations—including ours—remain provisional.

## 7 Conclusion

Stimulation parameters are the central determinant of what EES can achieve after spinal cord injury: electrode placement selects the functional target, frequency and mode distinguish facilitation from conduction block, and amplitude titration determines whether residual neural capacity is recruited or proprioceptive feedback is sacrificed. This narrative review mapped these parameters across every major outcome domain, and the map reveals a field of substantial contrasts—two reproducible motor paradigms (tonic configurations plus intensive training, and spatiotemporal programs), one nearly standardized autonomic indication (hemodynamic control), and several domains (pain, bowel, trunk control) where parameter evidence remains anecdotal. Above all, the evidence base is fragmented and weak: dominated by uncontrolled case series of *n* ≤ 10, contradicted at the trial level by the first adequately controlled (null) RCT, and complicated by unresolved questions of task specificity, EES–tSCS equivalence, discomplete anatomy, and the variable protocols and doses of co-delivered training.

For clinicians working today, a prudent position is defensible. Current evidence does not yet establish routine clinical indications, and EES is best regarded as an adjunct that amplifies residual pathways rather than a stand-alone restorative therapy; where it is used, it should be embedded in structured protocols or trials as one component of a combined stimulation-plus-training intervention. In that context, candidates may be stratified by imaging and physiology rather than clinical completeness labels alone; initial parameters may be selected from the scenario-specific ranges in Table 1 and then individualized through recruitment-curve–anchored titration; stimulation should be embedded in intensive, task-matched training; and expectations should be calibrated to the evidence level of each outcome domain (Table 2)—most confident for standing and blood-pressure support, most cautious for neuropathic pain.

For the field, the priorities are unambiguous: adequately powered sham-controlled randomized trials of epidural stimulation, designed around—rather than against—the blinding and training-dose constraints reviewed here; a mandatory, uniform parameter-reporting checklist to enable cross-study synthesis and replication; routine imaging- and electrophysiology-based stratification of participants, so that “responder” parameters can be linked to residual anatomy; prospective long-term safety registries; and explicit cost-effectiveness evaluation to inform equitable access. Only when parameter science is tested under controlled conditions will EES move from a collection of notable individual recoveries to a reproducible, evidence-graded therapy for spinal cord injury.
